# Reconstructing the directionality of coupling between cortical populations with negative phase lag

**DOI:** 10.1186/1471-2202-16-S1-P166

**Published:** 2015-12-18

**Authors:** Fernanda S Matias, Leonardo L Gollo, Pedro V Carelli, Mauro Copelli, Claudio R Mirasso

**Affiliations:** 1 Instituto de Física, Universidade Federal de Alagoas, Maceió, AL 57072-900, Brazil; 2 Systems Neuroscience Group, Queensland Institute of Medical Research, Brisbane QLD 4006, Australia; 3 Departamento de Física, Universidade Federal de Pernambuco, Recife PE 50670-901, Brazil; 4 Instituto de Fisica Interdisciplinar y Sistemas Complejos, CSIC-UIB, Campus Universitat de les Illes Balears, E-07122 Palma de Mallorca, Spain

## 

Understanding how information is processed in the brain is one of the key areas in neuroscience research.Different tools have been employed to reconstruct directional influence and to infer the effective connectivity between distinct brain regions. Particularly, it has been shown [1] that in non-linear delay-coupled oscillating systems exhibiting a negative phase lag, Granger causality (GC) might not provide the correct direction of information flow (from the driver to the receiver). Such systems have been studied before in the theoretical framework of Anticipated Synchronization (AS) developed in the field of dynamical systems [2]. This counterintuitive synchronization regime can be a stable solution of two dynamical systems coupled in a master-slave (driver-receiver) configuration when the slave receives a negative delayed self-feedback. Recently, it has been shown that unidirectional coupled neuronal population models can also exhibit AS [3]. In these cortical like populations the delayed feedback has been replaced by a dynamical inhibitory loop mediated by interneurons. Here we show that in these biologically plausible models, GC provides the correct directionality of the coupling for both positive and negative phase differences. In fact, when compared to experimental data in the primate cortex our model reproduces experimental phase lags, coherence spectra and GC spectra [3].
